# A Circle-Monitor for Computerised Assessment of Visual Neglect in Peripersonal Space

**DOI:** 10.1371/journal.pone.0082892

**Published:** 2013-12-12

**Authors:** Lena Ulm, Dorota Wohlrapp, Marcus Meinzer, Robert Steinicke, Alexej Schatz, Petra Denzler, Juliane Klehmet, Christian Dohle, Michael Niedeggen, Andreas Meisel, York Winter

**Affiliations:** 1 NeuroCure Clinical Research Center and Department of Neurology, Charité - Universitätsmedizin Berlin, Berlin, Germany; 2 Center for Stroke Research Berlin, Charité – Universitätsmedizin Berlin, Berlin, Germany; 3 Department of Biology, Humboldt University Berlin and Cluster of Excellence NeuroCure, Charité - Universitätsmedizin Berlin, Berlin, Germany; 4 Median Klinik Berlin-Kladow, Berlin, Germany; 5 Center for Rehabilitation Research, University of Potsdam, Potsdam, Germany; 6 Department of Educational Science and Psychology, Free University Berlin, Berlin, Germany; Centre de Neuroscience Cognitive, France

## Abstract

Current assessment of visual neglect involves paper-and-pencil tests or computer-based tasks. Both have been criticised because of their lack of ecological validity as target stimuli can only be presented in a restricted visual range. This study examined the user-friendliness and diagnostic strength of a new “Circle-Monitor” (CM), which enlarges the range of the peripersonal space, in comparison to a standard paper-and-pencil test (Neglect-Test, NET).

**Methods:**

Ten stroke patients with neglect and ten age-matched healthy controls were examined by the NET and the CM test comprising of four subtests (Star Cancellation, Line Bisection, Dice Task, and Puzzle Test).

**Results:**

The acceptance of the CM in elderly controls and neglect patients was high. Participants rated the examination by CM as clear, safe and more enjoyable than NET. Healthy controls performed at ceiling on all subtests, without any systematic differences between the visual fields. Both NET and CM revealed significant differences between controls and patients in Line Bisection, Star Cancellation and visuo-constructive tasks (NET: Figure Copying, CM: Puzzle Test). Discriminant analyses revealed cross-validated assignment of patients and controls to groups was more precise when based on the CM (hit rate 90%) as compared to the NET (hit rate 70%).

**Conclusion:**

The CM proved to be a sensitive novel tool to diagnose visual neglect symptoms quickly and accurately with superior diagnostic validity compared to a standard neglect test while being well accepted by patients. Due to its upgradable functions the system may also be a valuable tool not only to test for non-visual neglect symptoms, but also to provide treatment and assess its outcome.

## Introduction

Neglect is a unilateral attentional, representation-memory or intentional deficit [1] that cannot be attributed to sensory or motor impairment and is defined as the inability to identify, orientate or respond to stimuli located in the contralesional hemispace [2]. About 40% of right brain-lesioned and 20% of left brain-lesioned stroke patients initially suffer from neglect to different degrees [3]. Symptoms not only yield problems in daily life, but also interfere with rehabilitation success [4]. Therefore, quick and accurate diagnosis and early assignment to specific treatment is essential to improve recovery of patients.

Currently, a number of different standardised paper-and-pencil tests are administered to diagnose neglect symptoms, including the Behavioural Inattention Test (BIT) [5] and its German adaptation, the Neglect-Test (NET) [6]. Computerised tests have also become frequently used, e.g. the subtest for neglect in the Test Battery for Attentional Performance (TAP) [7] which requires the detection of a target stimulus on a heterogeneous background. However, the tests mentioned have been criticised for their low ecological validity, as the tests do not match the requirements of daily living and target stimuli are only presented within a narrowly confined visual range (single screen or piece of paper), so that the requirement for visual scanning is clearly reduced.

The first problem has been countered by recent developments in computer technology which allow implementation of more realistic tasks, i.e. by means of virtual reality scenarios. In addition, more sensitive tasks were developed by establishing computerised versions of well-established paper-and-pencil tests (for a review see [8]). For example, Fordell, Bodin, Bucht and Malm [9] developed a virtual reality test battery for assessment and screening of acute neglect, using a goggle system to simulate 3D-Vision and a computerised version of standard neglect tests. The test battery correctly assigned patients and healthy controls to the respective groups and the automatic assessment of reaction times enhanced the diagnostic strength of the system.

Independently from 3D-environment simulation, computer-based assessments allow quantifying additional parameters (e.g., reaction times or visual search patterns) not available in standard paper-and-pencil tests [8]. For example, Rabuffetti et al. [10] implemented a touch-screen system for testing visuo-motor exploratory skills in five patients with chronic neglect using a cancellation task, a frequently used assessment method in paper-and-pencil tests, which allowed calculating different performance indices, like neglect severity, response Latency Index (i.e. search speed; more precisely the median of time between two target detections) or a Crossing Index (i.e. an indicator for the degree of structure of visual exploration). The authors demonstrated that neglect patients significantly differed from healthy controls in their performance through lower accuracy and higher values of Latency, Crossing and Neglect Indices.

In the present study, we obtained these parameters from a computer-based assessment. We developed a new assessment tool: the “Circle-Monitor” (CM): The CM is a circular arrangement of eight touch-screens with a chair in the centre, in which the patient can potentially interact with a 360° environment. However, in the clinical study presented here, only a field of vision of maximum 225° was used (activation of five screens). We examined whether the extension of the visual range (compared to standard tests) affected the diagnostic value of the tests. We implemented a series of established and newly developed neglect tests (Star Cancellation Test, Line Bisection - both adapted from the NET subtests - Dice Task, and Puzzle Test). The major aim of this study was to investigate the user-friendliness of the system and to compare its accuracy and efficiency for the assessment of visual neglect with the currently used gold standard, the paper-and-pencil test NET.

## Materials and Methods

### Participants and recruitment

The study was approved by the local ethics committee of the Charité University Hospital, Berlin, Germany. To be eligible, patients had to be diagnosed with left-sided hemispatial neglect as assessed by means of the TAP [7]. The clinical sample included ten patients with unilateral neglect (4 women, 6 men, average age of *M* = 60 years, *SD* = 8 years, see Table 1). All patients had a first-time ischemic (*N* = 9) or hemorrhagic (*N* = 1) lesion in the right hemisphere and unilateral left-sided neglect. Patients were recruited through a rehabilitation clinic (Median Clinic, Berlin) and a local retirement center (Pro Seniore Residenz Vis à vis der Hackeschen Höfe, Berlin). Five of ten patients were tested in the sub-acute stage (i.e. 2–12 weeks post-stroke), the other five were in the chronic stage (i.e. >3 months post-stroke). Nine patients were right-handed (LQ = 100, 10^th^ right decile, measured by Olfield's scale [11] with LQ ≤48: right handed, LQ ≤−28: left handed), one was both-handed (patient 7: LQ = −20, middle).

**Table 1:** Demographic and clinical characteristics of the patient sample.

Group	Pt	M/F	Age	Post-onset (months)	NIHSS	Stroke etiology	V&TDS		CAV- screen		CAV - ET	NET score	
							L	R	L	R	% correct	1–8	all
SA	1	W	51	1.5	12	i	7	10	3	9	8.3	54.5	120.5
	2	M	52	1	2	i	10	10	8	10	0.0	72.5	157.5
	3	M	52	2	2	h	5	10	4	9	0.0	69.5	154.5
	4	W	54	1	10	i	1	10	4	10	58.3	52	129
	5	M	68	3	12	i	0	10	0	5	0.0	31	-
CH	6	M	59	53	7	i	8	10	10	10	75.0	71.5	-
	7	M	59	5	8	i	0	10	3	7	0.0	62.5	-
	8	W	64	25	8	i	6	10	10	10	100.0	79.5	166
	9	W	69	6	4	i	8	10	9	10	58.3	70	144.5
	10	M	72	11	7	i	9	10	10	10	75.0	68.5	150
	M		60	10.9	7.2		5.4	10	6.1	9.0	37.5	63.2	146.0
	(SD)		(7.8)	(16.5)	(3.6)		(3.7)	(0.0)	(3.7)	(1.7)	39.5	(14.0)	(16.1)
CG	M		68	-	-	-	10	9.9	10	10	88.3	78.0	167.5
	(SD)		(9.8)	-	-	-	(0.0)	(0.3)	(0.0)	(0.0)	(15.3)	(2.5)	(2.5)

*Note.* Groups: SA =  subacute, CH =  chronic, CG =  control group. Pt =  patient; M/F =  male/female. NIHSS: National Institutes of Health Stroke Scale. Stroke etiology: i =  ischemic, h =  hemorrhagic stroke. V&TDS: visual and tactile double stimulation. CAV screen: CAV visual field screening. CAV-ET: CAV extinction test. NET Score: for subtests 1 to 8 and for the whole test battery. Mean (M) and standard deviation (SD) given for patients and healthy controls.

Patients with comorbid hemianopia were excluded if lesions in the striate cortex became evident during magnetic resonance imaging (MRI, see below) and also based on the results of the Computer-Based Assessment of Visual Function (CAV) [12].

The healthy control group was comprised of 6 women and 4 men and participants were age-matched to the clinical sample, *U* = 26, *Z* = −1.82, *p* = .075). Nine controls were right-handed (LQ = 100, 10^th^ right decile), one was both-handed (LQ = −40, 1^st^ left decile).

Subjects in both groups were excluded if they met any of the following criteria: current affective disorder, epilepsy, claustrophobia, severe cognitive deficits (MMSE<20), severe restriction of right arm movements or serious difficulties in speech comprehension. Prior to study inclusion, patients and healthy controls provided written informed consent.

### Standard Neuropsychological Examination

All participants completed a comprehensive neuropsychological assessment that comprised the following tests: (a) test for extinction: manual simultaneous bilateral stimulation in the visual and tactile modality; (b) computer-based extinction test (CAV subtest): two triangles were presented simultaneously for 300 ms in the left and right visual field. Patients had to decide whether the orientation of the triangles was identical, or not (2 alternatives forced choice) [12]; (c) computer-based examination of the visual field (CAV subtest): A small white circle (0.2°) was presented for 100 ms on a dark-grey background in one of the four quadrants. Patients had to detect and localize the transient visual event. In each quadrant, six test stimuli were presented; (d) the paper-and-pencil test NET [6] which represents the current gold standard for the assessment of neglect. For comparison with the CM-subtests, four NET-subtests were taken into account: Star Cancellation Test, Line Bisection, Figure Copying, and Clock Drawing.

The analysis of these subtests was adopted to be comparable to the CM results: (a) Star Cancellation Test: overall performance was separated in performance in the left and right visual field (amount of detected stars); (b) Line Bisection: Performance was rated with scores ranging between −50 and 50 instead of 1 to 3 points; (c) Figure Copying: Performance was rated with up to 3 points per visual field instead of 1 to 3 points for the whole figure; (d) Clock Drawing: Performance was rated with up to 2 points on each visual field instead of 3 points for the whole figure. Overall NET-Scores were calculated based on the conventional analysis.

### Tests in the Circle-Monitor

#### Hardware

**Figure 1:**
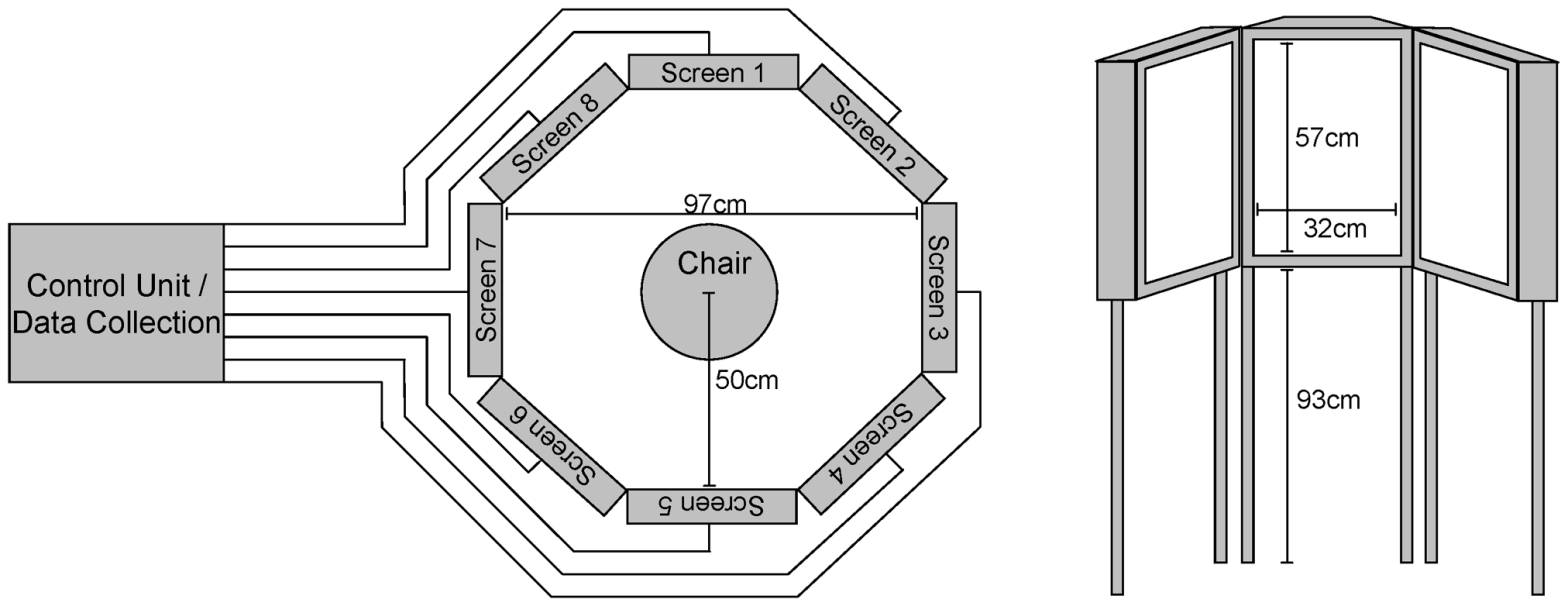
Schematical drawing of the CM.

The Circle-Monitor (CM) consists of eight touch-screens arranged in a circle (see Figure 1). A seat located in the middle of the CM can be accessed by swinging out two of the screens. Its 50 cm distance from the touch-screens is close enough to reach the touch-screens comfortably. The system is connected to a computer outside the CM. Participants completed tasks sitting inside the CM while an investigator controlled the testing session from a computer located in the same room and supervised subjects' behaviour by means of a video camera. Standardized instructions were given prior to the start of the CM sessions by the investigator who demonstrated the experimental tasks on the CM-screens while the CM-doors were open. Both, normal subjects and patients used their right hand (i.e. ipsilesional). For this proof-of-principle study, only one to five screens were active at a time. For a detailed description of the hardware see elsewhere [13], [14], [15], [16].

#### Tests

**Figure 2:**
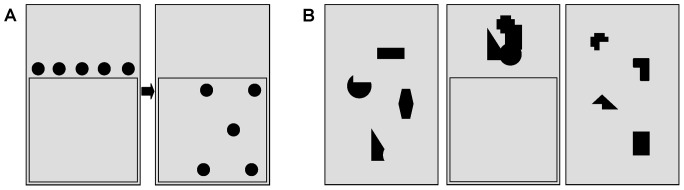
Layout of Dice Task (a) and Puzzle Test (b). In the Dice Task, patients were asked to use all dots and create a dice pattern. In the Puzzle Test, patients were required to select the correct pieces (from the left and right screen) to create the puzzle in the middle screen.

The total duration of the CM-assessment was 10–15 minutes. All participants completed four different tests in the CM:

a) Star Cancellation Test (SCT)
  The SCT was based on the Star Cancellation Tests from the NET [6]. Twenty large stars with a diameter of 3.5° were displayed at random positions on the five screens (screens 7, 8, 1, 2, 3 in Figure 1; 4 stars per screen) and surrounded by distractors in the form of small stars, letters and words. The task was to touch all the large stars as fast and as accurately as possible and to ignore distractor stimuli. The SCT was administered twice, hence 40 stars could be detected in total. Individual scores were calculated for each participant including a Neglect, Latency and Crossing Index [10] and the total number of marked stars in each visual field.
b) Line Bisection (LB)
  The LB was based on the Line Bisection from the NET [6]. Four 15° long horizontal lines were displayed at random positions on a single screen (screen 1). The task was to position a slider in the center of a horizontal line. The LB was repeated so that there were 8 trials in total. An average bisection position for each participant was calculated ranging from −50 to +50 (artificial unit: 1 unit  = 0.15°). A score of 0 therefore indicates symmetrical bisection, negative scores indicate bisections displaced to the left, positive scores indicate bisections displaced to the right.
c) Dice Task (DT)
  The DT was a simplified version of the Baking Tray Task [17]. Participants were asked to arrange large dots that were located at the top of one screen (screen 1) in a way that would represent dice spots (see Figure 2a). In total, six trials were completed, two each with four, five and six dots. For this task, deviation scores were calculated (Formula of the deviation index (dice task deviation, DTD): 
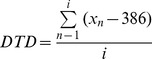
 with i =  number of dots, 386 =  vertical middle of the 768 pixel screen). A score of 0 indicates a perfect horizontal distribution of dots, negative score values indicated a shift to the left of a vertical middle line, positive score values indicated a shift to the right.
d) Puzzle Test (PU)
  The PU was based on the Hooper Visual Organization Test [18]. A figure consisting of four pieces was presented on the upper part of the middle screen (screen 1). On the screens to the left (screen 8) and right (screen 2) four different pieces were presented (8 in total, see Figure 2b). The task was to select the correct pieces (there were 2 correct ones on each side) and move them to the lower half of the middle screen, so that the figure could be reconstructed. The PU consisted of a series of five different puzzles. For each participant, the number of correctly selected pieces was calculated, separated into pieces of the left and right visual field, therefore 10 correct pieces could be selected on each side yielding a maximum score of 20.

### User-friendliness Rating

All participants were asked to answer a short rating scale (5-point Likert scale) after the last assessment in the Circle-Monitor. Ratings assessed the difficulty of the CM tasks and the experienced fun in comparison to the standard test, as well as the clarity of the tasks (intuitive comprehension) and feeling of security during the assessment (both in regards to the CM).

### Magnetic Resonance Imaging

Scanning was conducted using a 3-Tesla Siemens Trio MR-System at the Berlin Center for Advanced Neuroimaging. Individual lesion maps were manually delineated on the respective T1 images of individual patients. The latter were subsequently registered to MNI standard space using unified segmentation [19] as implemented in Statistical Parametric Mapping, SPM5 (Wellcome Department of Imaging Neuroscience, London, UK). The resulting normalisation parameters were used to register individual lesion maps to standard space and an average lesion image was created using Matlab® (The MathWorks, Natick, MA) which is shown as a surface rendering on a standard brain for visualisation.

### Statistics

Similar to the study by Rabuffetti et al. [10] a Crossing and Latency Index was calculated for each participant (only for the Circle-Monitor). The Crossing Index (CI, expressed as percentage score) is an indicator of the number of path crossings during the search of the stars divided through the total number of detected stars [10]. The total CI was calculated by adding up the CI of the first and second Star Cancellation Test trials.

Latencies (defined as the time lapse in seconds between current touch *T_i_* and the previous touch *T_1-i_*, *L_i = _T_i_ − T_i-1_*) were calculated for each participant [10]. The median of the Latency distribution for each participant was represented by the Latency Index (LI).

Using SPSS (Version 17) descriptive statistics were calculated for all assessed measures: (a) Star Cancellation Test: percent of correctly detected stars, CI, LI; (b) Line Bisection/Dice Task: deviation from the middle; (c) Puzzle Test: percent of correct among all selected pieces; (d) CopyTask/Clock Drawing: rating points.

Statistical differences were analysed using analysis of variance (ANOVA): In case of (a) SCT: a 2×2×2 ANOVA with the within-subjects factors *test* (Circle-Monitor vs. NET) and *hemispace* (left vs. right) and the between-subjects factor *group* (patients vs. controls); (b) LB: a 2×2 ANOVA with the within-subjects factor *test* and the between-subjects factor *group*; (c) SCT Latency Index, PU, Copy Task and Clock Drawing: each a 2×2 ANOVA with the within-subjects factor *hemispace* and the between-subjects factor *group*. Post-hoc comparisons were made by means of t-tests for independent (group comparisons) and independent (hemispace comparisons) samples. Group differences in SCT Crossing Indices and DTD (Dice Task Deviation) were tested by Mann-Whitney U-tests for independent samples. Degrees of freedom were corrected according to the Greenhouse-Geisser criterion [20]. The level of significance was *α* = .05 in all analyses.

In order to compare the discriminative value of the tests embedded in the NET and in the Circle-Monitor system, discriminant analyses were run additionally. In the first run, test results from the NET served as predictor variables, and in the second run, results from the Circle-Monitor system. The percentage of correct assignment of healthy controls and patients to their respective groups following the two runs was compared descriptively. For the user-friendliness examination, the positive and negative answers were analysed with regards to frequency.

## Results

### Magnetic Resonance Imaging

**Figure 3:**
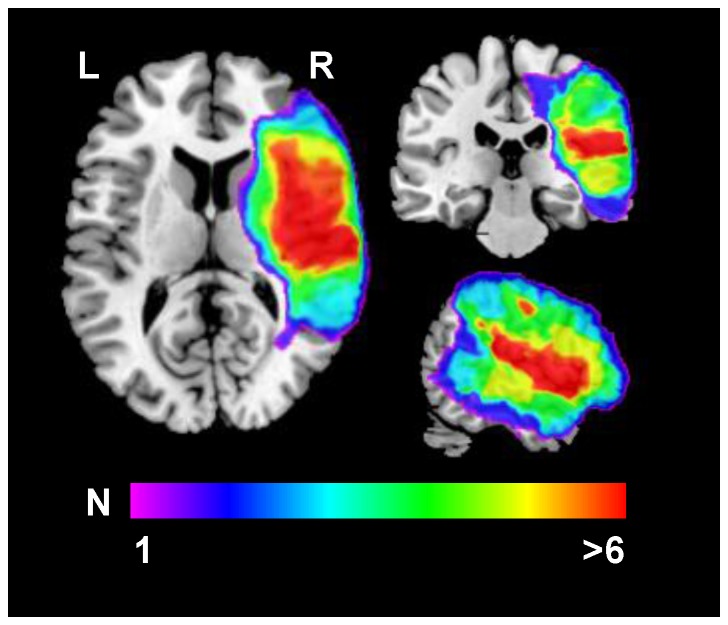
Lesion overlay plot (N = 9). Lesion overlap was highest in the right posterior superior temporal gyrus and insula as indicated by reddish colours, N: # patients with lesion, L: left, R: right, position of slices (MNI coordinates x/y/z): 54/-26/12.

**Figure 4:**
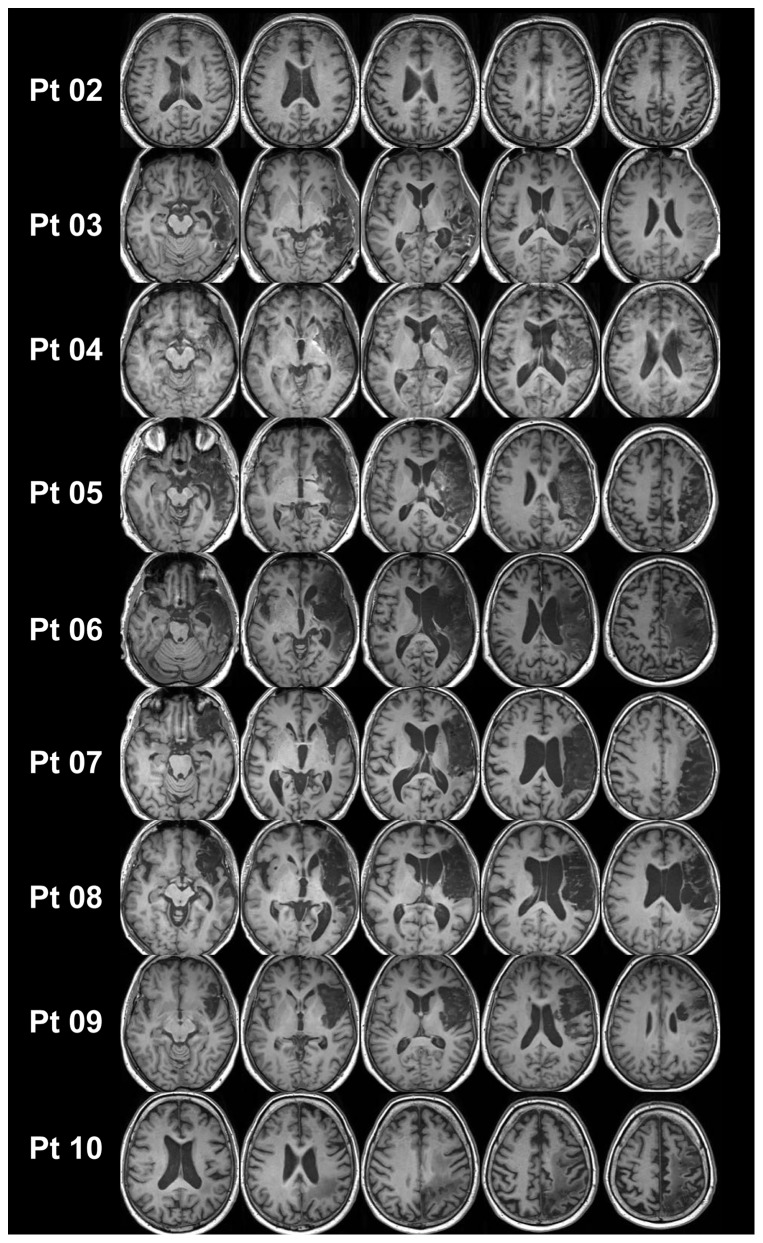
Lesion patterns of individual patients. Patient numbers correspond to patient IDs in Table 1 (patients #2-10). No MRI was available for patient #1. Right side of the brain corresponds to right hemisphere.

T1 weighted magnetic resonance imaging (MRI) scans were available for 9/10 patients (patient no. 1 met exclusion criteria for MRI). MRI confirmed unilateral lesions mainly in temporo-parietal and frontal regions of the right hemisphere in line with lesion locations frequently associated with neglect symptoms [21]. Lesion patterns of the patients are illustrated in Figure 3 as an overlay plot. Representative axial slices illustrating lesion extent in individual patients are shown in Figure 4.

### Standard Tests

In the visual and tactile double stimulation and the CAV subtests screening (CAV screen), patients performed worse than healthy controls on their contralesional (left) side (all *p*<.01, for all comparisons). Their performance on the ipsilesional side did not differ from the controls' performance. In the Extinction Test (CAV-ET) and the NET patients performed worse than healthy controls (*p*<.01, see Table 2).

**Table 2:** Results of the standard neuropsychological tests.

	Patients		Controls				
Test	N	M (SD)	N	M (SD)	T-value	Df	p
V&TDS	10	5.0 (3.8)	10	10 (0.0)	−4.340	9	.001*
	10	10.0 (0.0)	10	9.9 (0.3)	1.000	9	.341
CAV screen	10	6.1 (3.7)	10	10.0 (0.0)	−3.337	9	.009*
	10	9.0 (1.7)	10	10.0 (0.0)	−1.861	9	.096
CAV – ET NET Score	10	37.5 (39.5)	10	88.3 (15.3)	−3.792	11.64	.003*
	10	63.2 (14.0)	10	78.0 (2.5)	−3.295	9.55	.009*

*Note.* V&TDS: visual and tactile double stimulation. CAV screen: CAV visual field screening. CAV-ET: CAV extinction test. NET Score: for subtests 1 to 8 and for the whole test battery. N: Number of patients/controls. M: Mean, SD: Standard deviation. Df: Degrees of freedom. Stars (*) indicate significant group differences.

### Direct comparison: CM vs. NET

Both test batteries, CM and NET, included two standard tests usually applied in neglect diagnostics: star cancellation and line bisection. The first analysis was focused on these subtests.

#### Star Cancellation Test (SCT)

**Figure 5:**
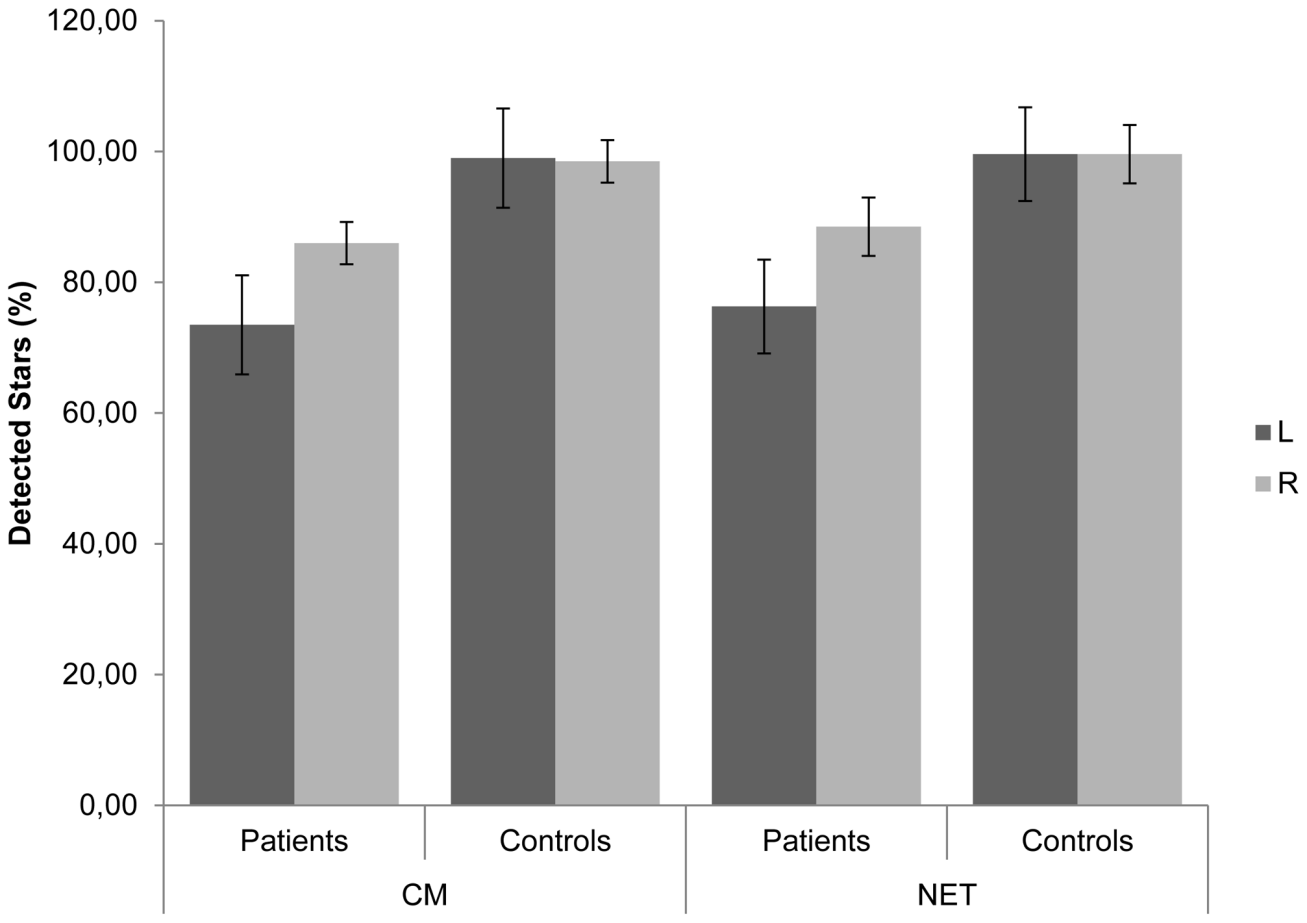
Star Cancellation Test: Percentage of detected stars, split into test-system, experimental group and visual field. The figure shows means and standard errors. Best possible performance (100%) in NET was 27 stars, in CM 20 stars per side. CM: Circle-Monitor, NET: Neglect-Test.

In the NET SCT patients showed poor performance with 76.3% detected stars on the left side (*SD* = 32.0) and 88.5% on the right side (*SD* = 19.9), whereas the healthy controls' performance was 99.6% detected stars on the left (*SD* = 1.2) as well as on the right side (*SD* = 1.2). Overall, patients detected 82.4% (*SD* = 25.1) and healthy controls 99.6% (*SD* = 0.8) of all stars (see Figure 5).Similarly, in the CM tests, patients detected only 73.5% (*SD* = 33.8) of the stars on the left and 86.0% (*SD* = 14.1) on the right side, whereas controls performed close to ceiling on both sides (left: *M* = 99.0%, *SD* = 2.1; right: *M* = 98.5%, *SD* = 3.4).

The 2×2×2 ANOVA with the within-subjects factors *test* (NET, CM) and *hemispace* (left, right) and the between-subjects factor *group* (patients, controls) confirmed that the controls performed significantly better than the patients (main effect *group*: *F*(1,18) = 5.943, *p* = .025, *η^2^ = *.248). However, this effect was significantly modulated by the factor *hemispace* (interaction *group* and *hemispace*: *F*(1,18) = 4.713, *p = *.044, *η^2^ = *.208) indicating that the performance was more impaired in the left hemispace in the patient group. Neither the main effect, nor the interaction was modulated by the factor *test*.

#### Line Bisection (LB)

In the NET-LB patients bisected the lines on average 5.9 (*SD* = 6.3) units to the right, the controls separated them at an average of 0.5 (*SD* = 1.0) units to the left of the centre. In the CM-LB results were highly similar with an average bisection of the lines 4.8 units (*SD* = 3.2) to the right of the middle in the patients group and 0.3 units (*SD* = 2.0) to the right in healthy controls. A 2×2 ANOVA with the within-subjects factor *test* (NET, CM) and the between-subjects factor *group* (patients, controls) showed that the *group* effect was highly significant, *F*(1,18) = 15.149, *p = *.001, *η^2^ = *.457, whereas the results were independent from the factor *test*, *F*(1,18) = 0.038, *p = *.847, or the interaction of *test* and *group*, *F*(1,18) = 1.081, *p = *.312.

### Discriminant Analysis

The correct assignment of the participants to the correct group (control vs. patients) on the basis of test results was examined using discriminant analyses. In order to compare the predictive value of the gold standard test (NET) and the CM system, we ran two independent analyses for the two test systems (see Table 3). In contrast to our first analysis, the discriminant analysis also considered the subtests only available in the single test batteries.

**Table 3:** Variables included in the discriminant analysis.

CM: (Explained variance: 66.1%, p = 0.003)	NET: (Explained variance: 43.9%, p>0.05)
Star Cancellation Neglect-Index (SCNI, left minus right omissions divided by total number of targets)	Star Cancellation Neglect-Index (SCNI*)
Line Bisection Deviation (LBD*)	Line Bisection Deviation (LBD*)
Puzzle Side-Difference (PSD*)	Copy-Task Side-Difference (CoSD, right minus left score*)
Dice-Task Deviation (DTD)	Clock Test Side-Difference (ClSD, right minus left score)

*Note.* CM: Circle Monitor. NET: Neglect Test. Explained variance: explained variance by the given predictors. Stars (*) indicate significant predictors in the discriminant analyses.

#### Discriminant analysis based on NET

**Figure 6:**
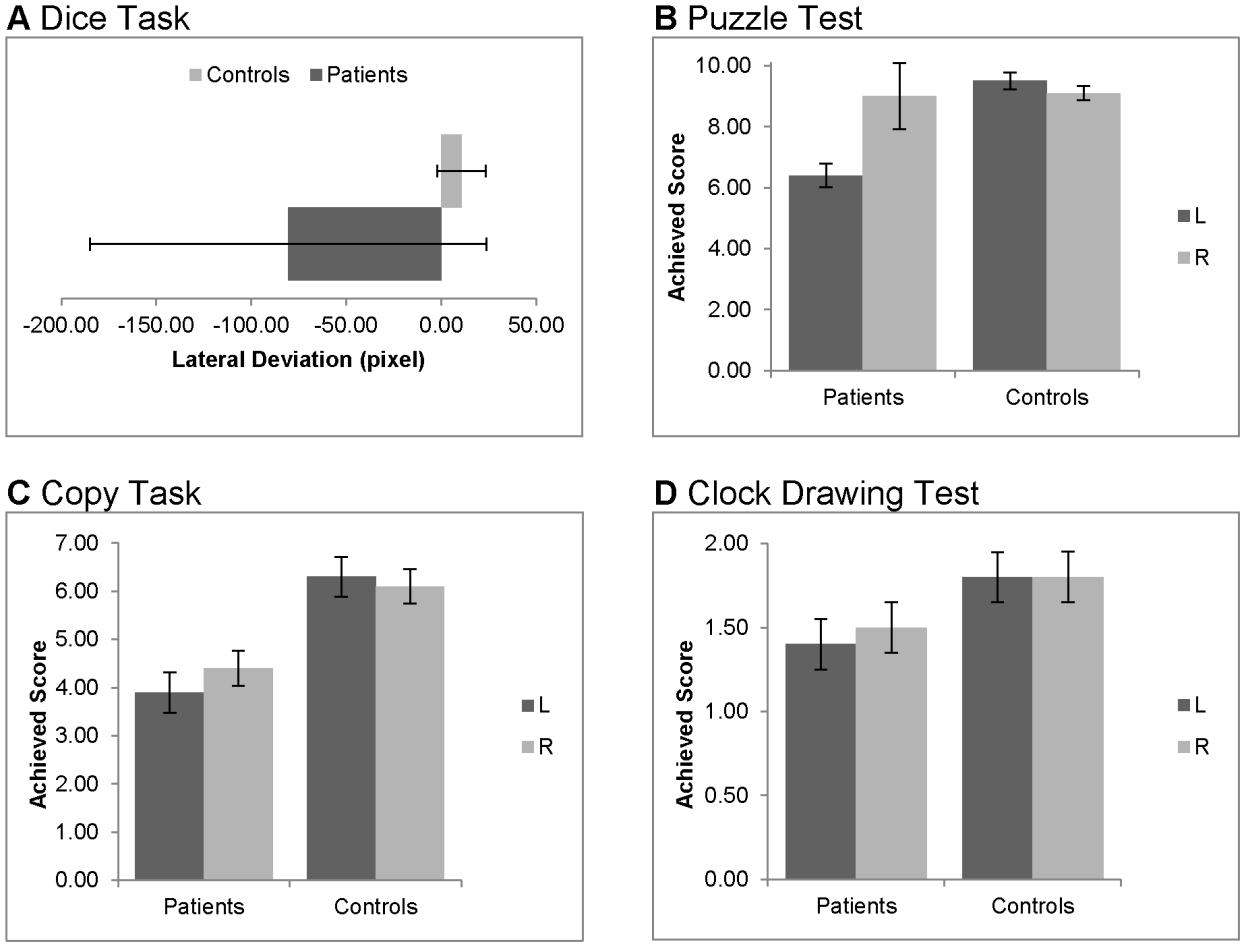
Results in visuo-constructive tests separated for patients and controls, left and right visual field. Dice Task (a) and Puzzle Test (b) are CM subtests, Copy Task (c) and Clock Drawing Test (d) are NET subtests. Diagrams show means and standard errors, L: left, R: right. Maximum achievable score per side: Puzzle: 10, Copy Task: 7, Clock Drawing Test: 2.

The analysis of the NET variables considered two additional visuo-constructive subtests, the “Copy Task” and the “Clock Drawing Task”. The results of both tests are shown in Figure 6c and d.

In the “Copy Task”, the ANOVA provided information that patients performed significantly worse that healthy controls (*group*, *F*(1,18) = 16.010, *p = *.001, *η^2^* = .471), but this effect was not modulated by the factor *hemispace* (interaction: *F*(1,18) = 0.491, *p = *.492). In the “Clock Drawing Task”, neither an effect of group assignment was found (*group*, *F*(1,18)  = 3.318, *p = *.085), nor an interaction of *group* and *hemispace* (*F*(1,18)  = .310, *p = *.584).

In the discriminant analysis, the performance in the subtests of the NET accounted for 43.9% of between group variability, and the discriminant function was not significant (*p = *.055). However, a detailed analysis revealed three significant predictors: Line Bisection Deviation (LBD; .842), Star Cancellation Neglect Index (SCNI; .567) and Copy-Task Side-Difference (CoSD; .471). Clock Test Side-Difference (ClSD; .148) was a poor predictor. Overall 90% of all subjects were correctly classified (80% of patients, 100% of controls). The cross validated hit rate was 70% (50% of patients, 90% of controls).

#### Discriminant analysis based on CM

The analysis of the CM variables also considered two additional visuo-constructive subtests, the “Dice Task” and the “Puzzle Test”. The results of both tests are shown in Figure 6a and 6b.

In the “Dice Task”, the mean performance (positive scores: deviation to the right, negative scores: deviation to the left) apparently indicates a clear deviation to the right in the group of patients. This difference, however, was not significant (Mann-Whitney-U-Test: *U* = 42, *Z*  = −.605, *p* = 0.579). In the “Puzzle Test” (n  =  9 patients), patients were found to select less correct pieces from the left hemispace than healthy controls (patients *M* = 7.1, *SD = *2.7; controls *M* = 9.5, *SD* = 0.7). A corresponding difference was not found in the right hemispace. Accordingly, the ANOVA indicated a significant interaction (*hemispace* and *group*, *F*(1,17)  = 6.825, *p = *.018, *η^2^* = .286). Post-hoc comparisons confirmed that the performance of patients and controls differed with regards to the left, *t*(17)  = −2.524, *p* = .033, but not to the right hemispace, *t*(17)  = −0.426, *p* = .676, see Figure 6c).

The discriminant function with the CM variables revealed a significant association between the groups and all predictors, accounting for 66.1% of between group variability, resulting in a significant discriminant function based on the CM (*p = *.003). A closer analysis of the structure matrix revealed only two significant predictors: LBD (.642) and PSD (.454), whereas SCNI (.295) and DTD (−.127) were poor predictors. The classification showed that overall 95% were correctly classified (90% of patients, 100% of controls). The cross validated hit rate was 85% (70% of patients, 100% of controls). When including the CI into the calculations, the cross validated hit rate increased by 5% to 90% (90% of patients, 90% of controls).

In sum, the discrimination based on the NET was inferior as compared to the CM.

### User-friendliness of the system

The user-friendliness ratings of patients and controls were similar: half of both groups rated the CM tests as more difficult than the standard NET, more than half of the patients and controls rated the CM tests as more fun than the NET (7/10 and 5/8 respectively). The majority of the participants rated the CM tasks as clear (patients 8/10, controls 8/8). All participants rated the CM assessment as safe (see Table 4).

**Table 4:** Rating of the CM assessment: Number of affirming responses.

	Patients	Controls
More difficult than NET	5/10	4/8
More fun than NET	7/10	5/8
Clarity	8/10	8/8
Safety	10/10	8/8

### Additional values obtained in CM testing

A previous study already indicated that computer-based assessment allows the registration of visual exploration pattern and response latencies (Rabuffetti et al. [10]). In order to validate these reports, we additionally measured the crossing index (related to visual exploration) and the response latency index in the CM subtest SCT.

The patients' crossing index (*M* = 27.0, *SD* = 12.1) was enhanced as compared to the controls (*M*  =  8.6, *SD*  =  6.5). This significant difference (*U* = 5, *Z* = −3.41, *p*<.001) indicated a much less economical visual search pattern in the patient group.

With respect to the response latency index (LI), one patient was excluded from analysis because he did not respond to stimuli in the left field. In patients, the LI was enhanced as compared to the healthy controls (patients: 1.6 s/star, controls: 0.9 s/star). This group difference was significant (*F*(1,17)  = 6.246, *p* = .023, *η^2^* = .269). The significant interaction between *group* and *hemispace* (*F*(1,17)  = 6.358, *p* = .022, *η^2^ = *.272) was due to the fact that in patients the latency was slightly increased when stimuli were presented in the left (*M* = 1.7, *SD* = 0.8) as compared to the right hemispace (*M* = 1.5, *SD* = 0.7). In sum, the additional variables exclusively delivered by a computer-based assessment are in line with previous results [10] confirming the sensitivity of the CM system.

## Discussion

### Summary of Results

In the present study, we provide first evidence that the newly developed CM is a safe and sensitive tool to assess visual neglect symptoms across a wide range of patients with acute and chronic neglect. In the CM subtests that are based on the gold standard (NET) the patients' performance was comparable to the NET subtests. However, the CM also allows a more detailed analysis of the time course of spatial exploration and provides additional values (CI and LI). The visuo-constructive tasks in the CM were more sensitive in the registration of hemispatial difficulties as compared to the gold standard. Accordingly, the correct classification of neglect patients following the CM tests is more accurate than the classification following the standard tests.

### Comparison with gold standard: Cancellation and bisection tasks

One of the main aims of this study was to investigate the diagnostic accuracy and sensitivity of the CM compared to the current gold standard (NET) to diagnose neglect symptoms. The NET or separate subtests have repeatedly been analysed for their validity and sensitivity [22], [23].

With respect to the standard version of the Star Cancellation Test (NET), results obtained in the CM were comparable. Both tests differed reliably between patients and healthy controls, and in both tests the omissions occurred more frequently in the patients' contralesional hemispace. Since the ratio between targets and distractors appears to be a crucial factor predicting the performance in patients with the performance being adversely affected by higher proportion of distractors [24], the detection rate would have been expected to be reduced in the CM (20 targets vs. 60 distractors) as compared to the NET (54 targets vs. 75 distractors). However, we assume that the reduction in item density resulting from the extension of the visual range in the CM compensates for the increase in target-to-distractor ratio.

Similarly to Rabuffetti et al. [10] the CM did also share the benefits of a computer-based assessment allowing the registration of the visual exploration pattern and response latencies. Our results also indicate a less structured visual search in neglect patients as reflected in an increased Crossing Index (CI). This effect is probably related to the finding that spatial working memory is reduced in patients with neglect [25]. With respect to the response latencies, data indicate that patients were slower in their visual exploration which might be due to a general decrease of work speed or due to the less structured visual search (represented by the CI). The patients' LI were slightly longer in the left than in the right hemispace, however, this difference was not significant in our patients. Rabuffetti et al.[10] found a latency gradient showing a less and less effective exploration when proceeding from the right to the left direction. This shows that visual neglect is not an on-off phenomenon but a *gradual* worsening of performance towards the left. Similarly, by means of a cancellation task, Line Bisection, and other tasks it has been demonstrated that neglect is not a completely lateralised phenomenon but that there is an almost linear decrease in the accuracy going from the right to the left side [26], [27]. Indices like the CI and LI might be able to identify milder forms of neglect in patients who show normal cancellation scores but impaired exploration performance towards the left hemispace [10]. Measurements for response times and search patterns may allow not only to initially diagnose neglect more accurately, but also help to identify the most appropriate approaches for treatment and to track minimal improvements in the patients' symptoms which might be overlooked by simple test-outcome analyses [28].

In the Line Bisection test, both NET and CM revealed highly significant differences between patients and controls. This confirms that Line Bisection is a valuable assessment method in neglect: Previous studies have shown that Line Bisection is probably more sensitive that drawing, cancellation and visual search tests [29], [30] and it is also related to the rating of neglect symptoms in everyday situations [31]. Since the CM Line Bisection task was administered on one screen only, its potential diagnostic value is probably underestimated. The optional usage of more screens could allow the examination of spatial gradients in the patients' bisection accuracy. Milner et al. [32] have shown that bisection performance in patients with neglect depends on the location of the lines in visual space, with lines presented on the left being bisected with a larger rightward bias than lines presented in the middle or on the right. Similar observations have been obtained other research groups [33], [34], [35], [36]. Milner et al. [32] suggested that for patients with neglect the left part of a line is perceived as shrunken relative to the right part, and that this distortion gradient increases from the right to the left hemispace. The results could also be explained by Small's [26] and Rabufetti's [10] findings that neglect is not fully lateralised but continuously worsens from the right to the left – thus producing an increasing leftward bias in Line Bisection. This could be further examined by the usage of an extended visual field in the CM Line Bisection.

### Comparison with gold standard: Visuo-constructive tasks

In the gold standard NET the copying and the clock drawing task indicated a difference in the visuo-constructive abilities of patients and healthy controls. At first sight, this finding substantiates the view that visuo-constructive tasks are valuable in neglect diagnostics [29]. However, the performance of patients did not differ between the hemispaces which indicated a more general deficit in visuo-construction than a hemispace deficit. One has to consider that impaired copying/drawing abilities are present in numerous neuropsychological deficits, such as apraxia or visual agnosia, and can be related to purely motor or proprioceptive or other disorders [37].

In contrast, a neglect-specific hemispace difference was obtained in a subtest of the CM system, the Puzzle task. In line with expected asymmetry, the Puzzle task revealed a deficit in the exploration of pieces presented in the left visual field, while there was no impairment in the ipsilesional field. The effect parallels earlier findings on manual exploration in neglect following right or left hemispheric lesions [38].

In contrast to the Puzzle Test, the Dice Task implemented in the CM system did not differ reliably between patients and healthy controls. This was surprising since the task shares the characteristics of the baking tray task (BTT) which is assumed to be a neglect test of high sensitivity [9], [39].

The lack of sensitivity of the Dice Task is probably due to the change in spatial arrangement: We used an established visual-spatial scheme – dice spots – in order to decrease cognitive demands. In previous studies, it has been reported that patients with cognitive impairment tend to place the cubes in other formations than instructed to [39]. However, it is possible that the choice of an established visual scheme also reduced the neglect symptoms: The relative position of dice spots is probably processed in a more global (distributed) visual processing mode, whereas the BTT requires a more local (focussed) processing mode. According to Peru & Chelazzi [40], patients with right-hemispheric damages and neglect symptoms are impaired in shifting attention to the contralesional field in a local rather than in a global processing mode.

### Sensitivity and acceptance

The discriminant analysis indicated that CM is slightly better in classifying patients and healthy controls as compared to the gold standard NET. As shown, the advantage of the CM can be further increased if additional variables (such as the Crossing Index) are considered. As suggested previously by Tsirlin et al. [8], implementing standard paper-and-pencil tests in computerised settings may lead to more accurate and more robust assessments due to the fact that they are (a) more independent of the administering person and that (b) additional parameters can be assessed. This assumption has been confirmed by using the CM system. Our results corroborate findings by Rabuffetti et al. [10] who also used additional parameters extracted from the computer-based assessment, i.e. the Latency and Crossing Index. Further studies will show whether an extension of the visual range in the CM system – here, only five out of eight monitors have been used – will also increase the clinical validity and sensitivity of the test.

Finally, the acceptance of the CM system has to be noted. The sensitivity of this test system has most likely also profited from the clarity of the instruction. Even more important, the patients – although placed in a semi-closed system (see Figure 1) – felt safe. Both factors contribute to the sensitivity of the CM system which was also accepted by participants with little computer experience and higher age.

### Summary: Benefits and limitations of the CM system

In line with other computerised assessment methods [9], [10] the CM system can identify patients with a unilateral neglect quickly and accurately while providing additional information not available from standard tests. The CM includes advantages of computerised tasks [9], [10], and in addition makes the examination of the full visual range possible.

The current CM hardware is already used elsewhere for assessment and training of other cognitive abilities, i.e. memory: a supermarket as a virtual reality environment run on the CM hardware [41], [42], [43], can be used effectively for the purpose of assessment and training of memory in healthy participants. The 360°-VR supermarket has also been tested on two patients with aphasia [44].

Despite of the promising data reported in this manuscript, we are aware that a reliable statement on the clinical validity of the CM system requires a considerably larger number of participants. Moreover, the CM system may not be usable by patients with severe motor impairments. The interaction with the touch screens would be hindered, thus, other means of testing would have to be established (e.g. control via eye movement/gaze tracking).

### Conclusions

In conclusion, the CM did not only identify neglect quickly and accurately but it was also widely accepted by our participants. It can therefore be seen as an efficient and sensitive tool for the assessment of neglect in stroke patients. Assessment with the CM is more detailed than the standard paper and pencil tests, as it provides additional information about reaction times (latencies) and detailed information about visual search patterns. The good usability of the system opens the way for application of the CM not only for assessment of visual neglect but also for training. The CM is a highly expandable system which is already used to simulate complex 3D-environments [13], [14]. Therefore, the CM offers the possibility to test for both, visual neglect-related deficits, but also for non-visual neglect symptoms. The latter symptoms have been described in several studies and might be underdiagnosed [45]. Furthermore, the CM has the potential to be used for diagnostics and training of other impairments in the domains of memory, attention, and perception.
